# The Evaluation of Surface Charges of Ascaris Lumbricoides, Giardia Duodenalis, and Taenia spp., in Preserved Fecal Samples Processed in Natura

**DOI:** 10.1007/s11686-026-01367-1

**Published:** 2026-09-11

**Authors:** Quéren Hapuque de Castro Novelli, Felipe Augusto Soares, Vinícius Salles Margatho, Enrico Piovesana Fernandes, Celso Tetsuo Nagase Suzuki, Edvaldo Sabadini, Bianca Martins dos Santos, Leyva Cecília Vieira de Melo, Amanda de Oliveira Baccin, Alexandre Xavier Falcão, Jancarlo Ferreira Gomes

**Affiliations:** 1 School of Medical Sciences, University of Campinas, São Paulo, Brazil; 2 ImmunoCamp Science and Technology, Vinhedo, São Paulo, Brazil; 3 Institute of Chemistry, Department of Physics-Chemistry, University of Campinas, São Paulo, Brazil; 4 Center for Parasitology and Mycology, Enteroparasites Center, Adolfo Lutz Institute, São Paulo, SP Brazil; 5 Institute of Computing (IC), Laboratory of Image Data Science (LIDS), University of Campinas, São Paulo, Brazil

**Keywords:** Parasitic intestinal diseases, Zeta potential, Diagnosis, Tropical medicine

## Abstract

**Objective:**

Parasite structures eliminated in feces have chemical characteristics resulting from their interactions with the environment in which they are found. These poorly documented physicochemical conditions may interfere with separation techniques used to detect helminth eggs and larvae, as well as protozoan cysts, oocysts, and spores. This interference negatively affects diagnostic protocols for these pathogens, which are generally low to moderate in sensitivity. As such, we sought to evaluate the surface charge by zeta potential (ZP) of the species *Ascaris lumbricoides*, *Giardia duodenalis*, and *Taenia* spp.

**Methods:**

We screened fecal samples that tested positive for these parasites at high intensity. These positive samples were then filtered through tubes containing 400 µm and 200 µm meshes, after which a 500 µL aliquot was diluted with 9 mL of deionized water and centrifuged (490 g / 5 min). This dilution and centrifugation procedure was performed 5 times to obtain a concentrated parasite sediment, which was then transferred to Petri dishes containing 7.5% formalin in deionized water. Eggs and cysts were visualized with stereomicroscopy equipment, captured with a 10 µL micropipette, and stored in Eppendorf tubes (2 mL).

**Results:**

Zeta potential values were analyzed with a Zetasizer nano device, and the average data obtained from in triplicate were − 28 mV, − 21.9 mV, and − 18.4 mV for *G. duodenalis*, *A. lumbricoides*, and *Taenia* spp., respectively.

**Conclusion:**

We conclude that the negative charges presented on the surfaces of these parasite structures in natural fecal samples can negatively affect laboratory diagnostic techniques.

## Introduction

Intestinal parasites are widespread worldwide and pose a high epidemiological risk in populations with social and economic vulnerability due to poor sanitation. The diagnostic techniques and medication types used to treat these parasitic infections are the focus of numerous studies aimed at informing public health policies to control these diseases with greater efficacy [1].

Numerous methods have been studied and applied for the separation of parasite structures, including cysts, oocysts, spores, larvae, and eggs, in routine laboratory diagnostics. The specific density of these parasitic forms is the physical factor most frequently addressed in the literature for improving concentration techniques. Due to their low cost and practicality, the most commonly used methods for separating intestinal parasites in routine laboratory diagnostics are spontaneous sedimentation and centrifugal sedimentation (using formalin, ethyl ether, or ethyl acetate). However, these methods do not account for the electrostatic forces and the inherent hydrophobicity of the parasite surface, which may reduce recovery efficiency and diagnostic sensitivity for these infectious agents [2]. Furthermore, few studies have examined separation based on egg surface characteristics, such as surface charge and hydrophobicity [3, 4].

The parasites' structures eliminated in feces possess external membrane characteristics that may interact with detritus and the surrounding fecal material. Hydrophobicity and surface charge, as characterized by zeta potential (ZP), are important factors in describing the interactions of the parasites with intestinal and fecal media. Some enteroparasite species have been found to possess negative surface charges in studies involving the ZP of environmental samples of *Giardia duodenalis* cysts and *Cryptosporidium* spp. oocysts under controlled conditions. Interactions between *Schistosoma mansoni* eggs and paramagnetic microspheres have also been observed to facilitate the isolation of these eggs for detection techniques [5]. Such interactions have also been observed during evaluations of the action of paramagnetic particles in capturing *Cryptosporidium* spp. oocysts [6]. When considering these interactions, one hypothesis is that *S. mansoni* eggs interact with the microspheres via electrostatic interactions between oppositely charged surfaces, which favor their adhesion [7]. Recently, a technique for separating and concentrating parasite eggs in feces has been proposed, utilizing a flotation mechanism. In this method, the issue of surface charge is fundamental, as the first stage of the process involves the use of carriers, which are molecules that, when adsorbed on the surface of the air bubbles, create a more favorable surface for capturing particles of opposite charge. As a result, parasites that attach to the bubbles rise and are concentrated at the surface [8].

In this study, we aimed to investigate the ZP values of eggs from two helminth species and cysts from a protozoan species, obtained from fecal samples preserved in buffered formalin after concentrating the parasite structures without the use of chemical reagents.

## Methods

### Sampling and Ethical Considerations

For this study, we used samples obtained at the Ouro Verde Hospital—Municipal Laboratory of Campinas—São Paulo, Brazil, with approval by the Comitê de Ética de Pesquisa da Universidade Estadual de Campinas (Unicamp Research Ethics Committee), approved under protocol number 79823117.7.0000.5404. After screening, positive samples were evaluated for infection intensity (low, moderate, or high) to select those with high egg infection intensity using the Kato-Katz method.

### Egg and Cyst Separation Technique

The samples were preserved in appropriate collection tubes containing 7.5% formalin. After screening by direct examination, 1500 mg of feces was filtered by centrifugation at 490 g using a tube with 400 µm and 200 µm meshes. After filtration, 1 mL of sediment was transferred to 10 mL tubes, whereupon 9 mL of deionized water was added to resuspend the samples before centrifuging (490 g) for 5 min. This process was repeated five times to eliminate small fecal debris and partially purify the sample for the concentration of parasite structures. After the final centrifugation step, two 500 µL aliquots of the sediment were transferred into 1 mL Eppendorf tubes.

**Fig. 1:**
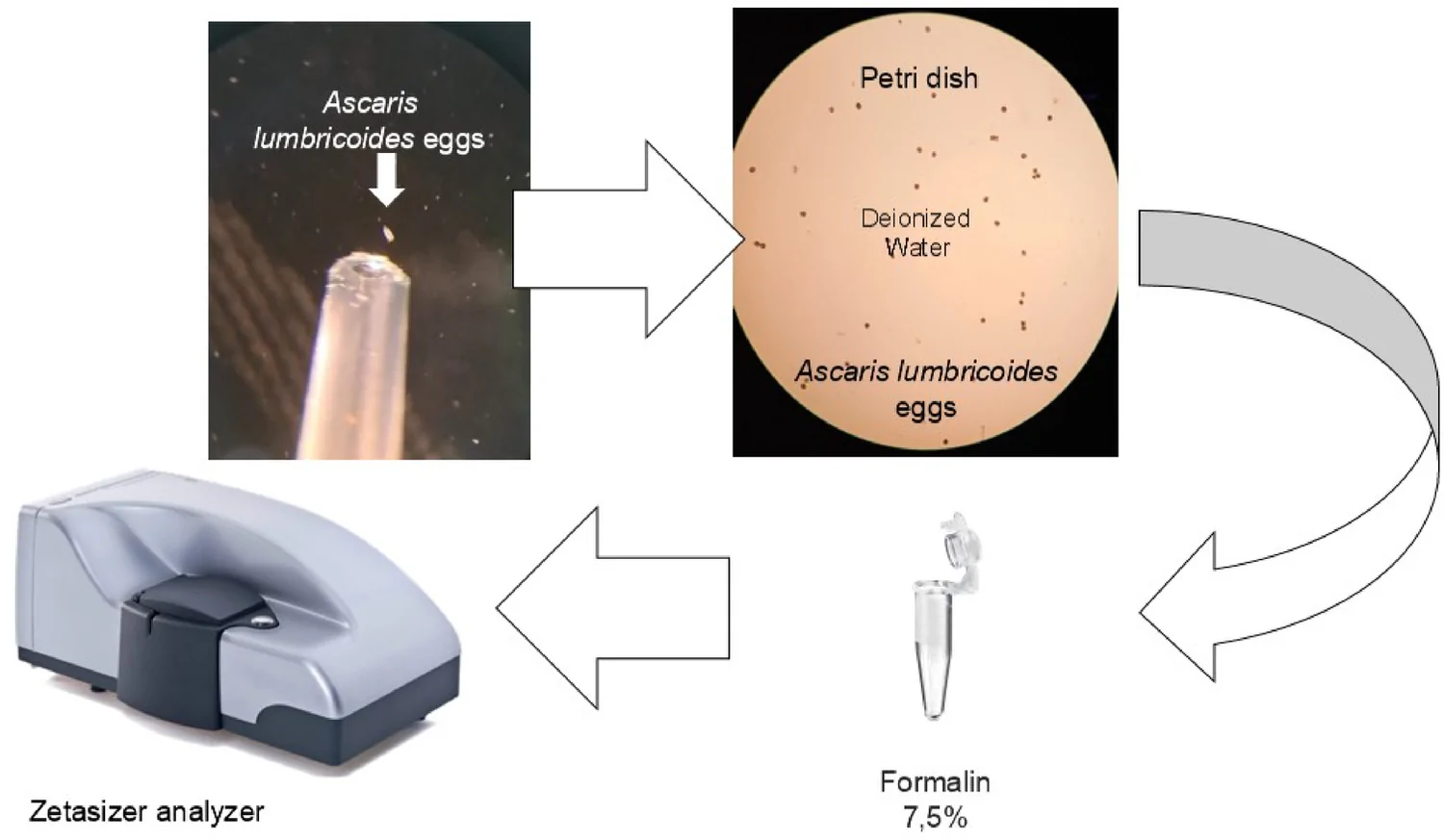
Procedural technique for the total separation of parasite eggs from filtered fecal sediment. Eggs were captured with a micropipette (10 µL) and transferred to a fresh tube for dilution in deionized water or formalin in a Petri dish

Two Petri dishes (4 cm diameter) were filled with either 5 mL of deionized water or 7.5% formalin, to which the 500 µL aliquots of fecal sediment were added. Eggs and cysts from the fecal samples were dispersed across Petri dishes and then imaged using a stereomicroscope (Olympus SZX16). These parasite structures were then captured using a graduated, 10 µL micropipette and transferred to a 2 mL Eppendorf tube. This capture-and-transfer procedure, repeated 6 times, yielded 400 eggs and over 1,000 cysts, all free of fecal debris (Fig. 1).

### Evaluation of Zeta Potential

The samples were placed into separate tubes according to parasite species, with three tubes containing 7.5% formalin. A Zetasizer nano ZS (Malvern©) was used to characterize the surface charge of each parasite species’ external membrane by measuring the ZP in millivolts (mV) (Fig. 1).

**Fig. 2:**
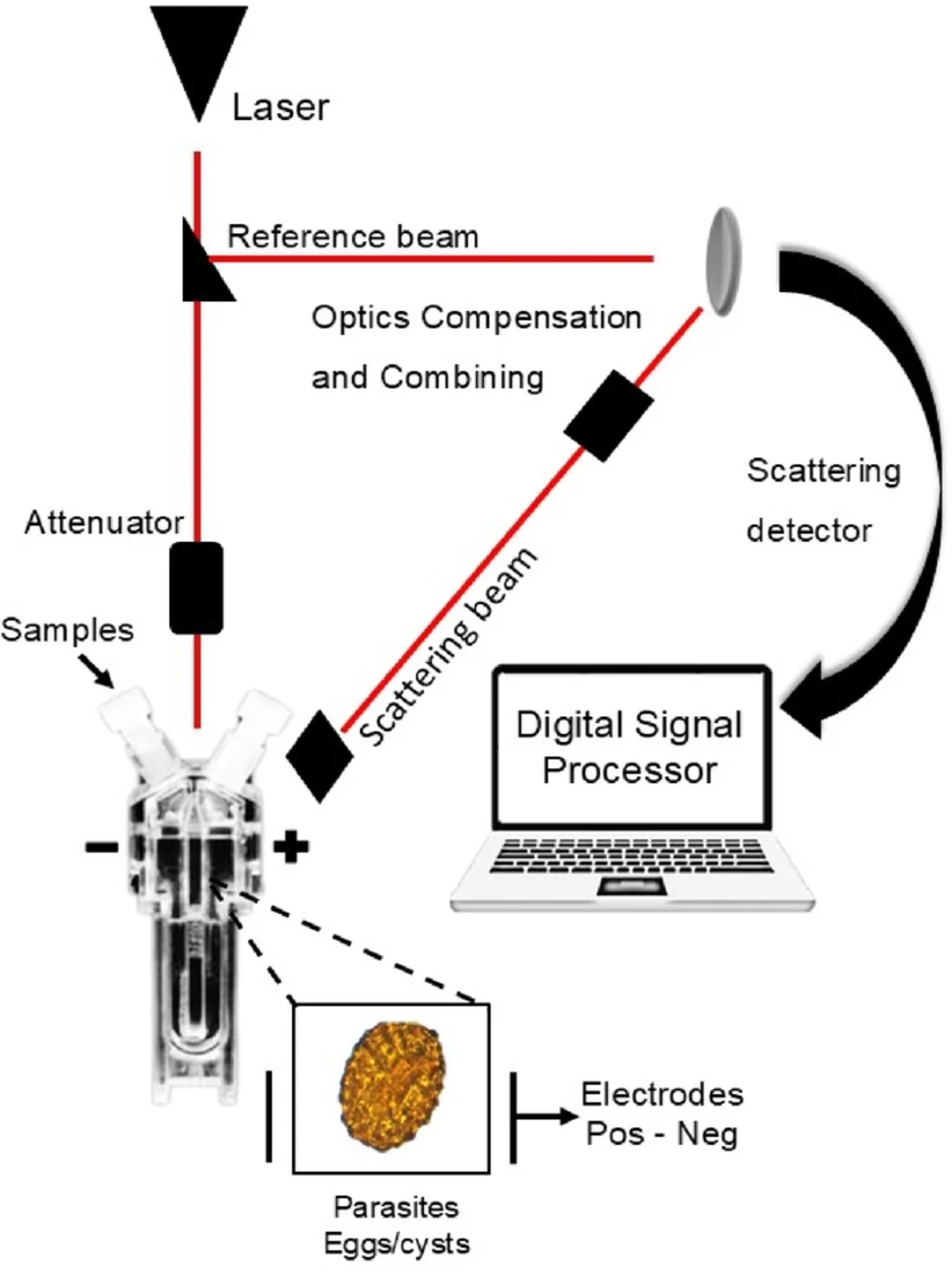
A diagram representing the elements of the equipment used to determine the electrical (zeta) potential on the surface of the particles (in this specific case, parasite eggs)

As schematically shown in Fig. 2, the zeta potential of dispersed parasite eggs is measured based on their electrophoretic mobility. An electric field is applied to the sample cell, inducing particle motion at different speeds. Particles with a higher charge density tend to move faster than less charged particles.

tk 2Electrophoretic mobility is determined by analyzing the Doppler effect in the frequency of laser light scattered by the particles. The frequency shifts observed are related to particle speed and can be used to measure electrophoretic mobility and zeta potential.

Each sample was evaluated in triplicate to determine test quality (good or undefined), which may vary due to factors such as sample concentration, bubble presence, viscosity, and conductivity.

### Statistical Analysis

The charge measurements (mV) obtained from the Zetasizer analyses are shown as the average and standard deviation of each sample's triplicate measurements. The data were then tabulated and compared across species groups using the ANOVA test in BioEstat 5.0. Differences were considered statistically significant using a p-value threshold of 0.05.

## Results

During screening, 42 samples tested positive for helminth and protozoan species. Of these, we selected three samples each of *G. duodenalis*, *Ascaris lumbricoides*, and *Taenia* spp.; these samples exhibited sufficient infection (classified as high during screening) for effective separation of parasite structures.

The surface charges measured for these species ranged from − 14.2 to − 32.1 mV, with *G. duodenalis* cysts exhibiting the most negative charge, averaging − 28 mV (Table 1).

**Table 1:** Measurement of the Zeta Potential (ZP) in millivolts (mV), in triplicate, of samples containing cysts and eggs of intestinal parasites preserved in a solution of formalin, 7.5%, shown with mean, standard deviation (Std Dev), and *p* values

Record	Sample name	ZP	Record	Sample name	ZP	Record	Sample name	ZP
		mV			mV			mV
1	Giardia (1)	− 25.3	1	Ascaris (1)	− 18.7	1	Taenia (1)	− 16.5
	Giardia (1)	− 25.7		Ascaris (1)	− 23.4		Taenia (1)	− 17.2
	Giardia (1)	− 27.5		Ascaris (1)	− 25		Taenia (1)	− 14.2
2	Giardia (2)	− 26.9	2	Ascaris (2)	− 16.8	2	Taenia (2)	− 16.4
	Giardia (2)	− 30.1		Ascaris (2)	− 16.4		Taenia (2)	− 19.5
	Giardia (2)	− 32.1		Ascaris (2)	− 17.9		Taenia (2)	− 17.5
3	Giardia (3)	− 29.3	3	Ascaris (3)	− 25.4	3	Taenia (3)	− 19.4
	Giardia (3)	− 28.5		Ascaris (3)	− 26.1		Taenia (3)	− 22.8
	Giardia (3)	− 26.5		Ascaris (3)	− 27.6		Taenia (3)	− 22.5
Mean		− 28.0	Mean		− 21.9	Mean		− 18.4
Std Dev		2.22	Std Dev		4.43	Std Dev		2.87
*p* value		< 0.0001	*p* value		0.0032	*p* value		0.0523

**Fig. 3:**
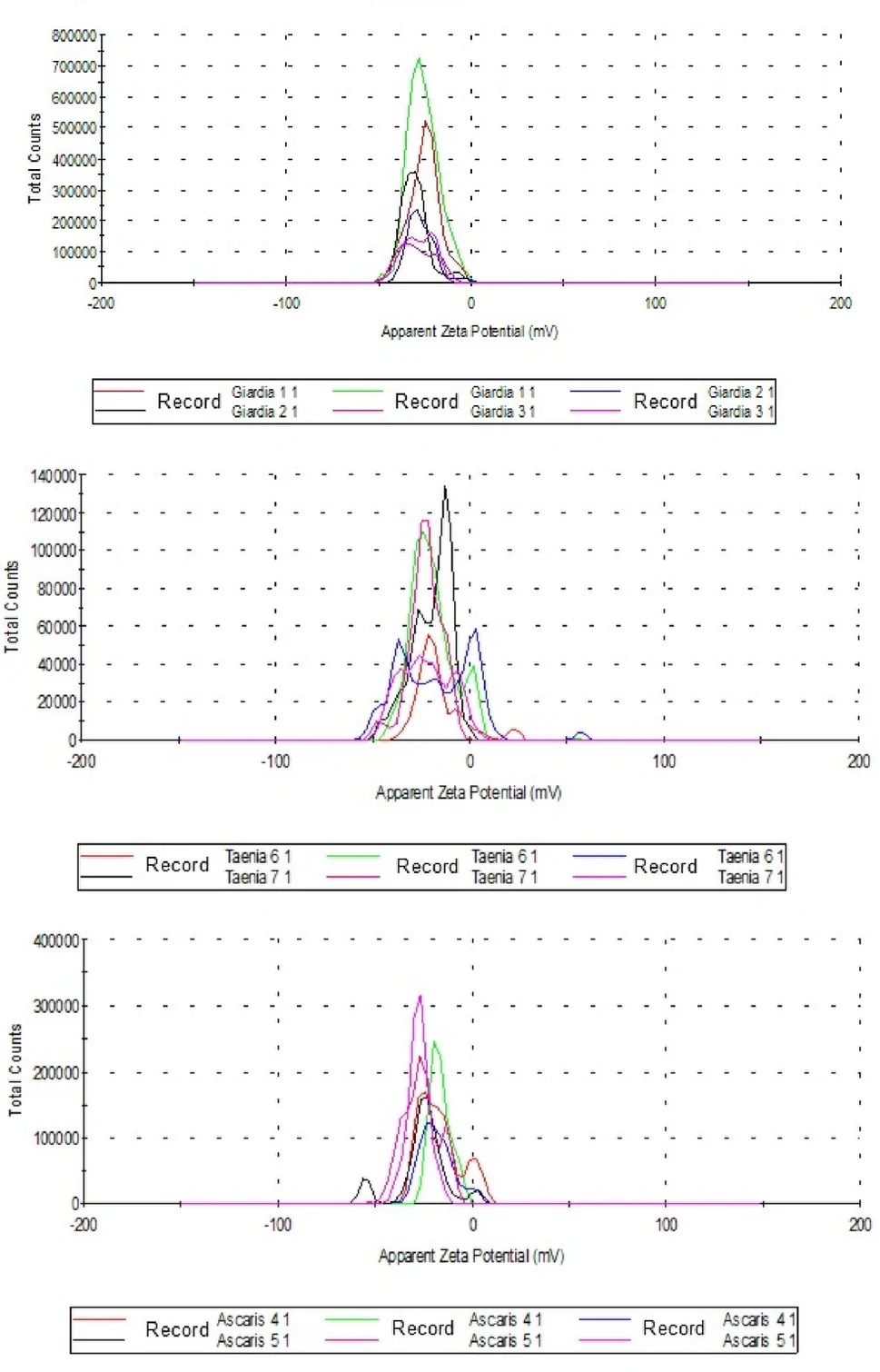
Distributions of apparent zeta potential in millivolts of the surfaces of the analyzed species

Three samples of each species (*G. duodenalis, A. lumbricoides,* and *Taenia* spp.) were evaluated, and two zeta potential (mV) records were obtained with Malvern Zetasizer software, version 8.02, as shown in Fig. 3. The software (Malvern©) classified all results as good quality.

## Discussion

In our study, we observed negative surface charges on *Taenia* spp. eggs and *A. lumbricoides* eggs, as well as *G. duodenalis* cysts, when these structures were separated without the use of any chemical reagents by manual pipetting. The ZP was then measured using a particle analyzer (Zetasizer). We also highlight that this is the first study to identify the surface charges of *A. lumbricoides* and *Taenia* spp. Eggs, which varied between − 14.2 mV and − 32.1 mV. A previous publication observed that the surface charges of *Cryptosporidium* spp. oocysts (− 38 mV) and Giardia spp. cysts (− 17 mV) were dependent on the medium pH, as shown by an increase in the ZP of these parasite structures with increasingly acidic pH [9]. In another study employing electrophoretic mobility at neutral pH, *Cryptosporidium* spp. oocysts and *G. duodenalis* cysts were found to have surface charges of − 40 mV and − 35 mV, respectively [10].

Our results also corroborate the negative surface charges observed on *Ascaris suum* eggs by Capizzi and Schwartzbrod (2001), who found that over 70% of these egg surfaces were hydrophobic [11]. The composition of *A. lumbricoides* eggs has been determined by electron microscopy, revealing an outer proteinaceous membrane, a middle layer composed of chitin, and a lipid layer; this may explain their hydrophobic nature [12]. Rosa et al. evaluated the use of an Aqueous Biphasic System (ABS), composed of solutions containing polymers and salts, to affect the physicochemical interactions with *A. lumbricoides* eggs. This process separates the eggs into the lower layer of the biphasic solution, indicating repulsive ionic interactions with the upper layer [13]. These electrostatic forces arising from ionic charges have not yet been clarified, as the parasite structures dispersed in a fecal suspension are generally subjected to varying conditions in chemical solutions (ether, ethyl acetate, zinc sulfate, sodium chloride, sucrose, etc.) used in fecal processing techniques [2].

In one publication regarding the standardization of a technique to separate helminths and protozoans from feces using the Formalin-Ether Concentration (FEC) procedure, Manser et al. [14] reported improved yield of parasites in sediment compared to previous studies by altering variables such as the g force and centrifugation time, as well as surfactants and solvents (ether or ethyl acetate). By doing so, some parasite species were found to be resistant to sedimentation, even when subjected to physicochemical forces that would typically cause them to sediment or float, suggesting the presence of a chemical force that acts against the movement of eggs, cysts, oocysts, or larvae. According to [15], differences in physicochemical properties among helminth eggs must be considered, as they may require adjustments to flotation and sedimentation techniques to optimize separation.

The techniques for parasite concentration that are widely used in the diagnosis of these pathogens use chemical reagents that can, at times, be aggressive to their morphology (saturated solutions of zinc sulfate, sodium chloride, and sucrose), or even damaging to the environment and the health of the people exposed to the volatile solutions (ethyl acetate, formaldehyde, and ether, for example) [2, 16]. These noxious reagents can be avoided by using ionic compounds that reduce the repulsive charges present in the fecal suspension. Tokumasu et al. [17] observed cytoadherence in microvascular endothelial cells, promoted by red blood cells (erythrocytes) infected with Plasmodium falciparum trophozoites, which exhibited a zeta potential of − 14.6 mV, significantly lower than that of uninfected cells.

Our results provided insight into the ionic forces that act on the surface of parasites (in natura), which can guide the reassessment of parasite concentration protocols in such a way as to use compounds that can reduce repulsive charges and favor a more effective concentration of parasite structures for diagnoses in clinical analysis and clinical pathology laboratories. Moreover, these data on surface charges can open the door to new studies on the synthesis of nanoparticles specific to enteric pathogens.

## Data Availability

No datasets were generated or analysed during the current study.
